# Exploring factors that influence the practice of Open Science by early career health researchers: a mixed methods study

**DOI:** 10.12688/hrbopenres.13119.2

**Published:** 2021-01-18

**Authors:** Ksenija Zečević, Catherine Houghton, Chris Noone, Hopin Lee, Karen Matvienko-Sikar, Elaine Toomey

**Affiliations:** 1 Faculty of Arts, Department of Psychology, University of Ljubljana, Ljubljana, Slovenia; 2 School of Nursing and Midwifery, National University of Ireland, Galway, Galway, Ireland; 3 School of Psychology, National University of Ireland, Galway, Galway, Ireland; 4 Centre for Statistics in Medicine, Nuffield Department of Orthopaedics, Rheumatology and Musculoskeletal Sciences, University of Oxford, Oxford, UK; 5 School of Medicine and Public Health, University of Newcastle, Australia, Newcastle, Australia; 6 School of Public Health, University College Cork, Cork, Ireland; 7 School of Allied Health, University of Limerick, Limerick, Ireland

**Keywords:** Open science, early career researchers, health research, research careers, meta-research

## Abstract

**Background: **There is a growing global movement towards open science and ensuring that health research is more transparent. It is vital that the researchers are adequately prepared for this research environment from early in their careers. However, limited research has been conducted on the barriers and enablers to practicing open science for early career researchers. This study aimed to explore the views, experiences and factors influencing open science practices amongst ECRs working in health research.

**Methods: **Semi-structured individual interviews were conducted with a convenience sample of ECRs working in health research. Participants also completed surveys regarding the factors influencing open science practices. Thematic analysis was used to analyse the qualitative data and descriptive statistical analyses were used to analyse survey data.

**Results: **14 ECRs participated. Two main themes were identified from interview data; Valuing Open Science and Creating a Culture for Open Science. Within ‘Valuing Open Science’, participants spoke about the conceptualisation of open science to be open across the entire research cycle, and important for producing better and more impactful research for patients and the public. Within ‘Creating a Culture of Open Science’ participants spoke about a number of factors influencing their practice of open science. These included cultural and academic pressures, the positives and negatives of increased accountability and transparency, and the need for more training and supporting resources to facilitate open science practices.

**Conclusion: **ECRs see the importance of open science for beneficially impacting patient and public health but many feel that they are not fully supported to practice open science. Resources and supports including education and training are needed, as are better incentives for open science activities. Crucially, tangible engagement from institutions, funders and researchers is needed to facilitate the development of an open science culture.

## Introduction

Open science aims to make research materials and results openly accessible and available to as a wide an audience as possible. Although there is no commonly accepted definition, open science is an umbrella term ( Fecher & Friesike, 2013) encompassing several areas, including open access, open data, open source and open reproducible research ( Pontika *et al.*, 2015). In essence, open science is about transparency and collaboration with all stakeholders throughout the whole research cycle, from conception and design to data production, analysis and dissemination. Opaque research limits our ability to build on existing research, leading to unnecessary duplication and research waste. It has been estimated that 85% of biomedical research resources have been wasted on flawed and non-transparent research ( Chalmers & Glasziou, 2009). As such, openness and transparency is particularly important for health research to maximise research efficiency and ensure optimum outcomes for patient care and health service delivery.

The importance of open science is becoming increasingly recognised on a global scale ( Fecher & Friesike, 2013). Open science has been recognised as a key priority for the European Commission, with a heightened focus on open access publishing and open data for Horizon 2020 funded projects ( Guedj & Ramjoué, 2015). As such, it is crucial that researchers are adequately equipped to navigate an open science landscape. However, despite its growing importance, researcher awareness of and engagement in open science activities remains suboptimal ( Morais & Borrell-Damian, 2019). A 2017 survey of 1,277 European researchers from a wide range of disciplines including natural sciences, social sciences, engineering and medical/health sciences found that the majority of respondents were unaware of the concept of open science ( O'Carroll *et al.*, 2017). This survey also found that early career researchers (ECRs) have less knowledge of open science policies and practices compared to senior researchers, but reasons for this were not explored. In addition, ECRs are often heavily involved in research data collection and analyses, but often have less autonomy for research decision-making ( Allen & Mehler, 2019; Farnham *et al.*, 2017). As such, the particular barriers and facilitators to practicing open science may well be different for researchers at the beginning of their research career than for those at a later stage, and warrant in-depth exploration.

Achieving a transition to open science practices within health research is complex, and requires a holistic approach to behaviour change that considers multiple individual, institutional and sociocultural level factors. Previous research has found that data sharing is influenced by individual factors, such as authors’ attitudes, past behaviours, perceived social norms and abilities, as well as broader factors such as journal data sharing policies and institutional supports and career incentives ( Kim & Stanton, 2016; Zenk-Möltgen *et al.*, 2018). However, many studies have focused on specific singular aspects of open science such as data sharing. Moreover, few studies have explored the perceptions, benefits and challenges of practicing open science among ECRs specifically, nor have they explored specific challenges and opportunities that may exist within health research. In addition, existing studies are predominantly quantitative in nature and many have not sought to qualitatively explore factors at a depth needed for gaining insight and understanding into this phenomenon. We aimed to explore the perceptions and experiences of open science for ECRs working in health research. We also specifically sought to explore the barriers, facilitators and factors influencing their practice of open science activities.

## Methods

### Ethical approval and study protocol

Ethical approval for the study was obtained from NUI Galway Research Ethics Committee [Ref no 19-Mar-31]. Written informed consent was obtained from all study participants prior to data collection. The study protocol is accessible at https://doi.org/10.17605/OSF.IO/PKREN ( Zecevic *et al.*, 2020). As the qualitative method had priority and no reporting criteria for mixed methods research exists, this study is reported in accordance with COREQ criteria ( *Extended data:* Appendix 1 ( Zecevic *et al.*, 2020)).

### Study sample and setting

Study participants were a convenience sample recruited from a two-day introductory training workshop on open science, which was held in NUI Galway (Republic of Ireland) in April 2019 for early career researchers funded by the Irish Health Research Board. Participants self-defined themselves as ECRs when registering for the event, with no restrictions placed on eligibility. There is no unanimously accepted definition of ECR, however previous research has defined an ECR as *‘one who is currently within their first five years of academic or other research-related employment allowing uninterrupted, stable research development following completion of their postgraduate research training’* ( Bazeley, 2003).

### Study design

We used a convergent mixed methods design to elicit a comprehensive and holistic understanding of the research question ( Creswell *et al.*, 2011). A convergent mixed methods design typically requires qualitative and quantitative data from different sources to be collected, analysed separately and brought together and integrated during the interpretation phase ( Creswell *et al.*, 2011). Participants provided quantitative data via questionnaires and were subsequently followed up with individual semi-structured qualitative interviews. In our study, participants had self-selected themselves into the two-day workshop, with another level of self-selection to participate in this study, therefore interviewees may not have been truly representative of the general population of ECRs. As such, the qualitative data provided comprehensive insights of their experiences and perceptions of open science, with the quantitative data used to further describe the characteristics, beliefs and experiences of this sample and to add further context to the qualitative findings. Although we also originally intended (as outlined in the study protocol) to integrate quantitative and qualitative data regarding the factors influencing open science practices using triangulation methods ( Farmer *et al.*, 2006), this was deemed largely unnecessary and uninformative upon completion of analysis for each dataset, as due to their semi-structured nature and conversational flow during interviews, not all interviewees spoke about specific survey items.

### Quantitative data collection

Participants completed a study questionnaire before and after the workshop. The content and structure of the questionnaire were informed by the 2017 European open science survey ( O'Carroll *et al.*, 2017). The pre-workshop questionnaire collected data on participant demographics, such as gender, age, discipline etc. Both pre and post-workshop questionnaires ( *Extended data:* Appendix 2 ( Zecevic *et al.*, 2020)) included closed and open-ended questions exploring the knowledge and awareness of open science components and initiatives among early career health researchers, as well as their perceptions of the barriers and facilitators influencing their practice of open science activities. As interviews were carried out after the workshop, only data regarding knowledge, awareness and influencing factors from the post-workshop questionnaire were used in this study as this was perceived to be more relevant for characterising the sample. Pre-post questionnaire data was reported separately as part of a workshop evaluation for the funders ( Toomey, 2019).

### Qualitative data collection

Individual semi-structured interviews were carried out either in person or by telephone to participant preference. Interview duration ranged from 13–34 minutes, with an average of 21 minutes in duration. All the participants were interviewed within three weeks after the workshop and interviews were facilitated by one or two members of the research team (KZ, CH). Interviewers may have been known to participants from attendance at the workshop, but were introduced during interviews as independent researchers with no bias or hidden interests in the topic. A topic guide was developed by an experienced qualitative researcher (CH) with input from KZ and ET, and used to structure the interviews ( *Extended data:* Appendix 3 ( Zecevic *et al.*, 2020)). The topic guide specifically explored participants’ understanding and experience with open science and their perceptions of barriers and enablers to practicing open science, with specific probes to enable deeper exploration of the topic in question. Interviews were audio-recorded and transcribed verbatim. Member checking of transcripts was not conducted due to time constraints and also due to evidence suggesting the benefits of member checking for verifying accuracy of transcripts may be relatively small ( Hagens *et al.*, 2009).

### Data analysis

Basic descriptive statistical analyses including percentage distributions and median calculations were used to describe the closed-ended questionnaire data. Data analysis was conducted using Microsoft Office Excel. Open-ended questions were analysed by coding answers using themes identified during the qualitative analysis, whilst remaining open to the potential for iterative generation of new themes.

Interviews were analysed using thematic analysis ( Braun & Clarke, 2006), which was facilitated within NVivo 12 qualitative management software. Thematic analysis is an inductive approach to analysis, thus moving beyond description into interpretation and “telling a story” of the data ( Clarke & Braun, 2018), p106). First, transcripts were read several times by one member of the research team (KZ). The researcher then coded statements and citations from the interviews into nodes. Each node was named according to the content of the citations it encapsulated. If a citation did not fit in any of the existing nodes, the researcher created a new node and named it appropriately. The coding was an iterative process, and the researcher went back and forth between the transcripts and the formed nodes, sometimes re-naming the node to give a better idea of its content.

Once this initial round of coding was complete, the NVivo file was sent to a second researcher (CH) to review the nodes. Initial themes were then generated and refined by the two researchers through iterative cycles of discussion and review. The final themes, subthemes and their descriptions were then reviewed by a third researcher (ET) alongside the transcripts. The role of this third researcher was as a ‘critical friend’, by offering critical feedback on interpretations of the data and encouraged reflexivity by providing a “theoretical sounding board” ( Smith & McGannon, 2018) p113).

### Rigour

A number of strategies were employed to ensure the study was carried out in a rigorous and transparent way. Firstly, as outlined above, the research team agreed the coding and theme development from the qualitative phase. This exercise is known as peer de-briefing and is used to ensure the data is represented fairly in the developed themes to minimise researcher bias ( Houghton *et al.*, 2013). Secondly, we created a codebook within QSR NVivo to demonstrate the dependability of our findings ( *Extended data:* Appendix 4 ( Zecevic *et al.*, 2020)). Using the coding query function, we were able to illustrate the density of coded references from each participant across all sub themes, emphasising that our findings were grounded in the data ( *Extended data:* Appendix 5 ( Zecevic *et al.*, 2020)). This strategy also enhances transparency without the privacy concerns of publishing raw transcripts ( Tsai *et al.*, 2016).

## Results

### Participant characteristics

14 out of the 40 workshop participants agreed to participate in the mixed methods study. Ten were female. Four participants had obtained a PhD in the previous 1–2 years, one was 6 years post-PhD, seven were undertaking a PhD at the time of participating in the study and two of the participants did not have a PhD. Participant demographics are further described in Table 1.

**Table 1.** Study participants.

Characteristic	N (%) of participants ( *N* = 14)
**Gender**	
Female	10 (71.4)
Male	4 (28.6)
**Age group, years**	
20 – 25	2 (14.3)
26 – 30	5 (35.7)
31 – 35	4 (28.6)
36 – 40	1 (7.1)
46 – 50	2 (14.3)
**Nationality**	
Irish	10 (71.4)
American	1 (7.1)
South African	1 (7.1)
Italian	1 (7.1)
Not specified	1 (7.1)
**Country of work**	
Ireland	13 (92.9)
Not specified	1 (7.1)
Type of researcher *	
PhD/doctoral candidate	7 (50)
Postdoctoral researcher	5 (35.7)
Research clinician/practitioner	3 (21.4)
Research area *	
Medical research	3 (21.4)
Basic science	2 (14.3)
Health sciences/allied health	6 (42.9)
Health psychology	5 (35.7)
Population health/public health services research	12 (85.7)
Nursing/midwifery	1 (7.1)
Other (specify) Psychology Participatory community-based research Environmental psychology	3 (21.4) 1 (7.1) 1 (7.1) 1 (7.1)
Work institution *	
University	12 (85.7)
Private/non-governmental research institute	1 (7.1)
Non-profit organisation	2 (14.3)
Joint academic/governmental organisation	1 (7.1)

*Some questionnaire questions allowed multiple-choice answers. Therefore, in some cases the sum of the numbers in the result tables exceeds the total number of participants.

Briefly, survey data identified that participants reported better knowledge of open science components like open access and open peer review than of components such as open data, open source, open notebooks, open education and citizen science. 77% of participants reported concerns over personal data protection and confidentiality, and the lack of institutional guidelines as barriers to data sharing, while incentivising and recognition of open science activities for career progression were identified as important facilitators. Over half of survey respondents believed there to be a lack of financial support and infrastructure within their institution for open science practices. Specifically, the lack of supports such as funding, training, time and institutional buy-in were identified by 92% of survey respondents as one of the most substantial barriers to their ability to practice open science. Further details of post-workshop survey findings are available in *Extended data:* Appendix 6 ( Zecevic *et al.*, 2020).

### Thematic analysis

We identified two main themes from the qualitative data: ‘Valuing Open Science’ and ‘Creating a Culture for Open Science’ with a number of subthemes also identified (summarised in Table 2). A ‘wordcloud’ created using QSR NVIVO queries to illustrate most commonly used words when participants talked about open science is provided in *Extended data:* Appendix 7 ( Zecevic *et al.*, 2020).

**Table 2.** Themes and subthemes identified during post-workshop interviews with key findings.

Themes	Subthemes with key findings
**Valuing Open** **Science**	**The ‘what’ of open science** • Participants perceived open science as broader than open access, i.e. openness across the research cycle, from before the study starts until after it finishes
	**The ‘why’ of open science** • Participants perceived open science to be important because it leads to better research which leads to better overall impact of research for patients and the public
**Creating a Culture for Open Science**	**Cultural and academic pressures** • Current academic culture is a key factor influencing open science practices - for example, career development being measured on grants/publication, with open science not valued as much ○ For early career researchers (ECRs) in particular, publications were viewed as particularly important, for example, for PhDs by publication ○ In contrast, many felt also that open science may be harder for more senior researchers who are already entrenched in the culture to change their ways • For ECRs, open science practices are influenced by senior supervisors and team members
	**Increased accountability and the challenges of transparency** • Increased accountability deriving from transparency was commonly identified as a barrier to open science as an ECR, for example, the fear of being exposed, making mistakes or data confidentiality breaches ○ On the other hand, participants felt that increased accountability was also good, for example, improving the peer review process and ensuring reviewers are more constructive, and improving the quality of the overall research • Nefarious practices, such as the fear of others misusing available data, or fear of having research ideas being taken from them (i.e. being ‘scooped’), were occasionally identified as a barrier
	**Striving to be open** • All identified the importance and need for more training and resources to support ECRs and all researchers, and the need for this to be integrated into existing systems and driven from the top, e.g. institutional buy-in

***Valuing Open Science***

This theme explores how ECRs define and perceive open science and its importance.

*The ‘what’ of open science*

Participants perceived open science as a broad umbrella term, encapsulating 'openness' across the entire research cycle, i.e. from before a study starts (for example, using pre-registration and open notebooks) until after it finishes (for example, with open access publishing and data sharing). In general, this openness equated to a broad sense of transparency, availability, fairness, and replicability and reproducibility. Most interviewees had only recently learned that open science was more than just open access publishing at the end of the research cycle, an insight many attributed directly to the workshop.

*“Before [the workshop] I really thought open science was just about open access publishing and maybe just sharing data, so putting some data up on the open science framework or those kind of things. So I hadn’t really thought about kind of how the whole process can be open from beginning to end.*“ (INT 1)

Overall interviewees felt they did not have much experience with practicing specific open science activities, but the experiences they had were predominantly positive. Most interviewees had pre-registered a study or published a study protocol and some of them were planning to do so at the next opportunity.

*The ‘why’ of open science*

Interviewees generally agreed open science has many benefits, with some less obvious than others. They were concerned that there is a lack of awareness of the benefits of open science. In general, participants felt that practicing open science behaviours led to better research. Ultimately, open science was perceived as the better, more ethical and impactful, way of doing research than standard or traditional approaches.

*“It’s about doing ethical research so that if we have open transparent ethical research then it can better inform whatever it’s supposed to inform whether it be health care etc. So it leads to better research being done fairly and then secondly it leads to more reproducible research so others can build on that research when they know exactly what you did...Good transparency and open research is the cornerstone of doing good research.”* (INT 10)

For example, participants felt that practices like protocol publication would enable others to see what researchers planned to do, in a timely and accessible way. This provided opportunities for peer-reviewers to spot potential errors at an early stage of the research cycle. Pre-registering a study or publishing a protocol also helped in the planning of the research and thinking more thoroughly about the adopted study procedures.

*“… I found publishing a protocol really helpful because it meant I dedicated time at the outset of a project, i.e. the systematic review to plan and think ahead about the methodology which I was going to use, what outcomes I was going to look at as opposed to it all happening as I was going along. So I think it helped increase the rigour and the quality of my research by doing my protocol...and spending time on the protocol and going through the peer review process with the protocol made it strengthen.”* (INT 10)

Open science also provided opportunities for collaboration between researchers, through sharing data and other research materials. Another advantage of open science identified by participants was the reduction of research waste and duplication. According to some interviewees, because of the transparency open science brings, it makes research more reproducible and gives the possibility to build-up from existing research.

*“I suppose that it is a more transparent way of working that builds the capacity of the research community so that they’re avoiding maybe duplication or where they want to build on maybe smaller research studies that have been done that it allows for knowledge transfer then.”* (INT 7)

Further, open science allows publishing results that otherwise might not get published (such as studies reporting data that do not support their hypotheses).

***Creating a Culture for Open Science***

*Cultural and academic pressures*

Overwhelmingly, the current academic culture and its according pressures were discussed by interviewees as one of the main factors influencing open science practices. Despite being at an early stage in their academic careers, interviewees already felt under pressure in the academic world. They experienced time pressure, and an expectation to be continuously publishing. Because of this, many activities, including those required for open science, proved difficult to incorporate into their already busy schedules:

*“Well I think the flip side of it is the timing to engage and find and network, as well, with others about open science on a day to day running of and teaching and administrating and writing and trying to engage in research. We have all got so many hats on us that unless you know there’s a little bit more protected time for I suppose advancing ourselves and our own knowledge in certain areas.*” (INT 14)

Open access journals, interviewees reported, tended not have high impact factors which could be off-putting when trying to achieve career progression. Furthermore, some interviewees believed there was a perception that publishing in an open science journal or platform was easier and therefore would not be valued as highly for career progression as publishing in a traditional high impact journal:

*“I think there’s this perception, not among everyone, but there’s this perception that it’s not as…I don’t know if valid is the word, but it’s not seen as valuable that way. You know if it’s open science I think there are people that think that it’s easier to get accepted if it’s open science.”* (INT 6)

Some interviewees felt that pressures on ECRs were unique due to their unique position in the research world. Most were currently undertaking a PhD or working as a post-doctoral researcher for a supervisor or principal investigator. Interviewees felt that ECRs typically do not manage their own grants and have less autonomy in decision-making regarding activities such as practicing open science. Some felt that if an ECR decided to engage in open science, then they may not get support from the wider research team:

*“Whereas as an ECR it’s very much left to you. And if you’re the only person on the team who wants to do it then you’re the one who has to do it.”* (INT 1)

This was a problem especially if the opinions of their supervisor or more senior colleagues differed from their own. Interviewees reported that despite the wish to carry out open science activities, they had to prioritise other activities in order to advance their career.

*“I think at the end of the day, like, obviously we all have to whether we like it or not, there comes a point if you continue in research that you have to pay attention to those goals you have to hit to get promoted.”* (INT 6)

Interviewees identified a resistance to change in the current culture, some particularly among their more senior colleagues. Interviewees reported that more senior colleagues may not see the value in changing an established system that works, that they are used to and where they are also able to delegate certain tasks they may not see as important. However, at the same time interviewees felt the academic pressures and many challenges to doing open science were similar regardless of being at an early or more advanced career stage:

*“So I think in terms of challenges around knowledge and training I believe that they would also be challenges if not more so a challenge for more senior career researchers. So I think that’s definitely similar as well. Publications, impact factor, I don’t think things like that slow down as you become more senior....I think challenges are similar and probably all at the same level of knowledge I’d say as well and expertise and experience in doing this.”* (INT 12)

*Increased accountability and the challenges of transparency*

The concept of increased accountability was discussed by many as a key factor influencing the practice of open science by ECRs, both as a barrier and a facilitator. For example, open science is inherently transparent, which is valued, but this increased transparency also created several concerns for interviewees. The fact that others can see their work led to concerns about feeling exposed, and somehow vulnerable and open to criticism. They also had concerns about potential mistakes being identified:

*“And I know that’s something that you shouldn’t really be scared of because you know we’re all just kind of working and doing our best. But that would definitely be something that would be in the back of my mind if I was putting up my data that someone would rerun it and say you did this all wrong.”* (INT 1)

However, on the other hand, many also felt that open science practices such as open peer review would also make reviewers accountable and encourage them to be more thorough and diplomatic in their feedback to authors.

*“I think it probably creates efficiencies in the system because if you’re the named reviewer you probably would respond quicker. And if you know your information’s up there, you’re probably more likely to be pleasant at least and courteous with your colleagues. And at least you can see conflicts of interest more clearly as well.”* (INT 13)

In addition to the desire to protect themselves, interviewees also expressed a strong need to protect others, for example, research participants. A breach of confidentiality was considered of great importance, particularly when dealing with qualitative data and clinical data. The protection of research participants was an important factor influencing how interviewees felt about the practice of sharing data. They felt that openly sharing data was a key concern especially when treating rare conditions or narrow, very specific groups of people. Interviewees had legal, ethical and personal concerns around sharing data and making data accessible to anyone. They felt that there was still room for improvement and further consideration regarding how to achieve a balance between data sharing and confidentiality:

*“So it’s trying to make a balance, I think, between being open in your research and being ethical I suppose as well in that you need to protect your participants. So it’s kind of a balance I suppose of the two. So I thought that was quite interesting learning more about how people do that in open science as opposed to the kind of more traditional closed science.”* (INT 4)

The issue of nefarious practices came up briefly in some interviews, e.g. a fear of others misusing available data, or research ideas taken from them (i.e. being ‘scooped’). For example, one participant spoke of a fellow ECR colleague who felt their research plan had been stolen from them due to it being available publicly, and that it was important to have a way of safeguarding against that:

*“I suppose as well like I talked about earlier just kind of safeguarding that you know if you do do it* [make research plan available]. *I’d be completely happy to do it but I just think this idea of kind of scooping and taking people’s ideas, that you know, that the original person doesn’t suffer because of that.”* (INT 8)

*Striving to be open*

Participants identified many challenges to practicing open science. However, interviewees spoke also about what would help them to engage in open science more often, more willingly and more confidently. They felt that a change in university research culture was needed, starting at ECR stage or even earlier, at an under-graduate level. They felt that a research culture with open science at its core was needed so that open science activities would be encouraged and rewarded. This would enable research to be done in a collaborative and cooperative way, with wider audiences and researchers, and in turn lead to more impactful and applicable research:

*“And then I think … if academic institutions are encouraged to work more collaboratively with other sectors outside of academia they’re more likely to embrace the spirit of open science because I think it probably, you know the worries and the concerns that they would have from academia about you know are they publishing enough, you know are they hitting high level journals, all of those kind of pressures, if they could balance that by saying yes we’re working with open science and we’re actually doing very innovative research with collaborators who are outside of academia … so we’re actually producing really new and great research with wider applications.”* (INT 7)

Overall, however, a key requirement discussed was a shift in how university metrics are considered. Interviewees felt that a key incentive to practice open science would be the valuing or requirement of open science activities for career promotion and hiring criteria. Specific suggestions included recognising open science activities in a dedicated section of curriculum vitae, as a way to demonstrate alternative forms of impact:

*“And then as I mentioned unfortunately as an early career researcher you are focused on how many grants you win, how many publications you have. I think it needs to be recognised if on your CV that you’ve mentioned that you have your OSF platform and you have all this data. I think there needs to be a way of kind of acknowledging … that as well, the work that you’ve done through that.”* (INT 12)

One specific barrier discussed by participants was the lack of funding specifically for open science activities. They identified some solutions for the funding issue, such as internal funding from universities to reward the quality of pre-registered studies:

*“The issue for me particularly has been paying for the open access because in the instances where I publish open access I didn’t have specific funding allocated...So that’s a big barrier is just because we really wanted to have this open access but because of the money we might have chosen to go for a non-open access as well. But that was it. The money was the issue really.”* (INT 2)

Other aspects that would help support and develop an open science culture were discussed, including publishing regulations that would enable tracking and reporting of potential deviations from a pre-registered report. The creation of more policies around open science in general were also suggested, such as with a particular focus perhaps on sharing data. Another suggestion was to have an individual in academic settings responsible for open science; like an “open science officer” who could raise awareness, provide support and organise training events:

*“And so I would say one other possible help would be if universities actually instituted either an open science officer or something along that lines that [sic] promoted open science as opposed to just open access within a university, particularly amongst early career researchers and would hold regular workshops etc.”* (INT 5)

Overall, the need for broader structure and support was very present among the interviewed ECRs. Support for open science needs to come from academic institutions but also from funding agencies, and the wider research community. In addition, there is a responsibility for researchers themselves to start having conversations about the importance of open science. Learning from colleagues could encourage ECRS to practice it also:

*“I think if you see your colleagues doing it as well it’s kind of okay, you know, because it kind of encourages you to do the same. And I do feel like if you have to teach it that gives you kind of a bit of encouragement. I just think well if you’re preaching this you should be doing it also.”* (INT 13)

Social media and technology, more broadly, seemed to play a very important role in facilitating open science. Interviewees spoke about the value of specific publishing platforms in connection to open science (e. g. OSF platform, F1000 and HRB Open Research), as well as about blogs or tweets they came across. They also identified a benefit of technology for getting support for practicing open science:

*“I think there’s a lot of great information online. Even the likes of social media and companies like F1000 and OSF. Yeah I think that’s the best way to go and kind of look into who’s behind those committees whether it’s people from the HRB or high up researchers in the universities. And maybe even make contact with them... saying how can I go about doing this or what resources would you recommend.”* (INT 6)

Training and education was considered by interviewees as critical in promoting open science:

*“I was not aware before going to the workshop of even all of the resources that are out there online like Github.... Or even the ways that journals support you doing that in some instances and don’t in others. So definitely knowledge is a huge thing. … there’s this whole world out there that I didn’t realise. It’s huge. But again it’s just kind of within its own kind of group. …So, kind of, sharing that knowledge I suppose kind of knowledge translation stuff and making it a bit more accessible to people of different levels I suppose at undergrad or postgrad or something like that.”* (INT 4)

Participants spoke about helpful events, related to open science to raise knowledge and awareness around it, but underlined the importance of events focused on training. They had a lot of issues and questions around “how to do open science”, where can one pre-register a study, where can one share their data and how, where and how can one access materials of other researchers. Some interviewees felt that this information should be included in undergraduate and postgraduate curricula. They also felt that events should target as a wide an audience as possible:

*“I think it would be nice to involve as much of a wide audience as possible. So if it is research support officers, I mean the research offices at institutions, they can really help to create structure around open science. So it would be nice I think. It would be useful I think to raise their awareness because some of them might not be as aware as we want them to be.”* (INT 2)

## Discussion

Our study is one of the first to explore the experiences, perceptions of and factors influencing open science practices among ECRs working in health research. In general, participants identified a need for strong value in open science activities across the research cycle. However, they highlighted a number of factors influencing their ability to practice open science behaviours, including pressures generated by the current academic incentive structure and the research culture within their immediate work environment, and the positives and negatives of increased accountability. Interviewees emphasised the importance of structures and supports such as training and resources for facilitating open science practices, as well as co-operation across the research community including with other researchers, funding bodies and the wider community to establish an open science culture. The need for institutional engagement and career metrics that align with open science principles were also highlighted as crucial to incentivise and normalise open science practices.

ECRs have a particularly important role to play in the future of research. Recently described as the ‘harbingers of change’ ( Nicholas *et al.*, 2019), ECRs have the potential to influence and transform future research practices ( Stürmer *et al.*, 2017). However, ECRs experience a number of challenges to practicing open science, which may be heightened due to their circumstances. In our study, participants particularly highlighted the pressures already being faced to publish in order to establish their career. Although the issue of ‘publish or perish’ is by no means a challenge unique to ECRs, it is perhaps particularly problematic during a stage where the development and refining of research skills and the process of learning itself is crucial, and potentially more important than the final output. This is also the point at which we are socialised to the scientific practices that we will continue throughout our careers ( Felt *et al.*, 2013). Moreover, our study found that practice of open science behaviours and activities by ECRs is influenced by their supervisors, senior colleagues and attitudes towards open science behaviours within their immediate work environment. This further emphasises the importance of an all-inclusive, comprehensive approach to facilitate open science practices.

However, as well as the challenges faced, participants also highlighted specific opportunities afforded to ECRs by open science, such as improved research quality, increased visibility, dissemination and impact and enhanced opportunities for developing collaborations. For example, the greater availability and accessibility of research data and outputs increases the potential for developing new collaborations ( Modjarrad *et al.*, 2016), while registered reports offer a path to publication irrespective of findings ( Allen & Mehler, 2019), which may help ECRs meet the publication demands placed upon them, whilst concurrently embracing open science principles. As participants in our study pointed out, this increased transparency and visibility brings both advantages and disadvantages for ECRs. Despite seeing the benefits, ECRs also admitted feeling exposed and vulnerable in publishing their research materials and data, and a fear of being spotted making mistakes. In addition, participants discussed significant concerns and mixed feelings in relation to data sharing and making patient data available. Our findings on this point differ from Nicholas *et al.* (2019), who found ECRs across a wide range of disciplines were mostly positively inclined towards open data; however, this may be due to the fact that our study population were health researchers, and may identify issues of particular relevance for clinical research with rare conditions or smaller populations.

The concept of an ‘open science culture’ is an important tenet within this study. In particular, current academic culture and a lack of career incentives to practice open science are seen as critical factors influencing ECR behaviours. The lack of incentives has been previously suggested as a key challenge to open science for ECRs ( Allen & Mehler, 2019), reinforced by findings from Nicholas *et al*. (2019) who identified the existing reward system as detrimental to open science behaviours by ECRs. According to participants in our study, practices or systems that reward open science behaviours are rare and open science involvement is often not formally recognised, and sometimes discouraged. While the availability of funding, training and education events and resources was identified by ECRs in our study as vital for facilitating open science at a more basic level, participants also largely spoke about the need for cultural change and a shift in institutional reward systems towards valuing open science practices on a more complex level. However, a recent survey of European universities showed that while change had begun, research publications and grant funding were still the two main activities most incentivised and rewarded by universities. The report also commented that *‘no matter how hard advocates strive, Open Science will never be achieved unless accompanied by a change in the way researchers are evaluated'* ( Morais & Borrell-Damian, 2019) p6).

### Implications for Practice, Policy and Research

It is clear that appropriate support systems are needed to help ECRs to become the new generation of researchers and develop a culture that embraces open science. For example, our participants emphasised the importance of the availability of training events, education and resources within research active institutions to improve open science awareness, knowledge and skills for researchers. However, both formal and informal undergraduate and postgraduate research training and supervision in these institutions should also endeavour to include a holistic focus on the bigger picture beyond knowledge and skills, e.g., by focusing on the benefits and impact of transparent and better quality health research on patients and society, in essence to intrinsically motivate ECRs to practice open science behaviours. Moreover, both ‘top down’ and ‘bottom up’ approaches are needed from all stakeholders including research funders, university and institutional management and individual researchers at all levels (e.g. principal investigators, supervisors, ECRs) to facilitate the development of a research culture of transparency and openness. Specifically, as advocated for by Moher and colleagues, research active institutions and governing bodies should revisit the policies and metrics by which research and its outputs are measured, and accordingly how researchers are hired, promoted and evaluated and to explicitly seek engagement in open science activities ( Moher *et al.,* 2020). To support this, institutions could consider the appointment of specific open science officers or champions, or establishing open research committees that consider beyond just open data or open access publishing, but the entire research cycle, as well as building an open research culture.

For ECRs, our study highlights some substantial challenges to practicing open science, but with several opportunities also identified for ‘bottom up’ solutions and strategies to overcome and address these. First, ECRs can ‘get active’ by seeking out others interested in practicing open science and building an open science community through grassroots initiatives like ReproducibiliTea journal clubs, or Open Science Cafés, and build themselves a platform to be heard. Second, ECRs can ‘get vocal’ by using these platforms to lobby and engage with institutional management like ethics committees around data sharing, libraries regarding open access and open publishing policies, and university promotion committees. Third, ECRs can ‘get inquisitive’ by conducting research on research that would facilitate and promote open research practices, such as addressing sensitivities of data sharing and identify evidence-based ways of balancing ethics with data transparency. Further research is also needed to explore, develop, implement and evaluate the impact of different initiatives and interventions, such as the Framework for Open and Reproducible Research Training (FORRT), Facilitate Open Science Training for European Research (FOSTER) ( Pontika *et al.,* 2015) and Principles and Practices of Open Research: Teaching, Research, Impact, and Learning (PaPOR TRaIL) ( Egan *et al.,* 2020), at different levels (e.g. undergraduate, postgraduate, postdoctoral) to help overcome the obstacles to open science faced by ECRs working in health research and across other disciplines and improve open science practices.

### Strengths and weaknesses

This study has some limitations. It is important to note that our interviewees were recruited from participants of a two-day open science training workshop based in Ireland, and also volunteered to participate in interviews. This implies that our study sample is a sub-sample of the wider ECR target population who had a pre-existing interest in open science and may with, and who may also have been pre-equipped with awareness and deeper understanding of open science challenges and opportunities. Taking part in the workshop inevitably also influenced their knowledge about open science, which should be taken into consideration when interpreting the study findings. However, this also means that participants were in a position to provide substantial depth and richness of insight into an under-explored area of research. As such, these findings will provide useful comparison data for future similar studies or replications amongst other samples of ECRs. The rigour with which we conducted data collection and analysis also enhances the transparency and transferability of our findings.

Ironically, as a team of ECRs, challenges we encountered with making data from this study available mirror that of the study findings. Given the size and unique nature of our sample, de-identification of qualitative study transcripts was not deemed possible. However, as outlined in our methods section, we used NVIVO coding queries as advocated by Tsai *et al*. (2016) to provide a clear audit trail and facilitate transparency ( Houghton *et al.*, 2013). In addition, although de-identification of the quantitative data was deemed more easily achievable, the nature of our ethics approval meant that explicit consent for access to data beyond the research team had not been obtained. On reflection, this was deemed mostly due to a lack of forward planning for data sharing, with priority given to data confidentiality and participant protection. As such, our experience highlights another example of the need for better training and awareness for researchers in collaboration with other bodies such as ethics committees and data protection offices to facilitate appropriate data sharing.

## Conclusion

Our study aimed to explore the views and experiences of engaging with open science amongst ECRs working in health research. ECRs see value in open science and recognise its importance. They see benefits of open science as increased transparency of the process and improved research quality in general. However, they fear the visibility of potential errors and sometimes experience according feelings of vulnerability. Furthermore, many ECRs feel that they are not fully supported to practice open science, and that more education and training is needed, as well as incentives for open science activities. Crucially, tangible engagement from all stakeholders including institutional management, funders as well as individual researchers including ECRs themselves is needed to facilitate the development of an open science culture and improvement of open science practices.

## Data availability

### Underlying data

Given the size and unique nature of our sample, de-identification of qualitative study transcripts was not deemed possible. As outlined in our methods section, we used NVIVO coding queries as advocated by Tsai *et al*. (2016) to provide a clear audit trail and permit the running of coding and matrix queries to facilitate transparency ( Houghton *et al.*, 2013) (see *Extended data*). The data cannot be shared via an alternative route of closed access, since ethics approval did not provide explicit consent for access to the data beyond the research team. For the quantitative data, the information sheet given to participants clearly stated that their data would not be shared by anyone other than the research team. Aggregated results are provided in *Extended data.*

### Extended data

Open Science Framework: Open science study 2019; factors for practicing OS by ECRs ('Exploring factors that influence the practice of Open Science by early career health researchers: a mixed methods study'), https://doi.org/10.17605/OSF.IO/PKREN ( Zecevic *et al.*, 2020).

This project contains the following extended data:

- Pre and post-workshop questionnaires (Appendix 2)
- Topic guide for interviews (Appendix 3)
- Codebook within QSR NVivo (Appendix 4)
- NVIVO coding queries (Appendix 5)
- Post-workshop survey findings (Appendix 6)
- Wordcloud (Appendix 7)

### Reporting guidelines

Open Science Framework: COREQ checklist (Appendix 1) for ‘Exploring factors that influence the practice of Open Science by early career health researchers: a mixed methods study’, https://doi.org/10.17605/OSF.IO/PKREN ( Zecevic *et al.*, 2020).

Data are available under the terms of the Creative Commons Zero "No rights reserved" data waiver (CC0 1.0 Public domain dedication).
