# Supplementary material for: Exploring factors that influence the practice of Open Science by early career health researchers: a mixed methods study
Source: HRB Open Res. 2021 Jan 18;3:56. Originally published 2020 Aug 21. [Version 2] doi: 10.12688/hrbopenres.13119.2 (PMC7836032; doi:10.12688/hrbopenres.13119.2)
Supplement: Peer review history

# Peer review: Reviewer response for version 2

**DOI:** 10.21956/hrbopenres.14368.r28659

**Published:** 2021-01-22

**Authors:** Joshua Polanin

**Affiliations:** 1 Research & Evaluation, American Institutes for Research, Washington, DC, USA

The authors have sufficiently addressed my - and the other reviewer's - concerns. I appreciate the authors attentiveness to the issues.

Is the work clearly and accurately presented and does it cite the current literature?

Yes

If applicable, is the statistical analysis and its interpretation appropriate?

Yes

Are all the source data underlying the results available to ensure full reproducibility?

Partly

Is the study design appropriate and is the work technically sound?

Yes

Are the conclusions drawn adequately supported by the results?

Yes

Are sufficient details of methods and analysis provided to allow replication by others?

Yes

Reviewer Expertise:

Quantitative methodology; meta-analysis; open science practices.

I confirm that I have read this submission and believe that I have an appropriate level of expertise to confirm that it is of an acceptable scientific standard.

# Peer review: Reviewer response for version 2

**DOI:** 10.21956/hrbopenres.14368.r28660

**Published:** 2021-01-19

**Authors:** Emily Arden Close

**Affiliations:** 1 Department of Psychology, Bournemouth University, Bournemouth, UK

The authors have addressed my comments and I have no further comments to make.

Is the work clearly and accurately presented and does it cite the current literature?

Yes

If applicable, is the statistical analysis and its interpretation appropriate?

Yes

Are all the source data underlying the results available to ensure full reproducibility?

Partly

Is the study design appropriate and is the work technically sound?

Yes

Are the conclusions drawn adequately supported by the results?

Yes

Are sufficient details of methods and analysis provided to allow replication by others?

Yes

Reviewer Expertise:

Qualitative methodology; Mixed methods.

I confirm that I have read this submission and believe that I have an appropriate level of expertise to confirm that it is of an acceptable scientific standard.

# Peer review: Reviewer response for version 1

**DOI:** 10.21956/hrbopenres.14227.r28492

**Published:** 2020-12-07

**Authors:** Emily Arden Close

**Affiliations:** 1 Department of Psychology, Bournemouth University, Bournemouth, UK

This extremely well-written paper is very timely. As the authors state, with the growing global movement towards open science it is important to ensure researchers are prepared for it from early in their careers. Although the findings are not representative of the general population of ECRs, the finding that even ECRs who had a keen interest in open science felt they were not fully supported to practice it is extremely valuable for identifying ways this issue can be overcome. The qualitative research in particular was conducted with a high level of rigour. 

 My only criticism is that the suggestions for how to address the barriers mentioned in the article are not very specific. For example, the authors state: “It is clear that appropriate support systems are needed to help ECRs to become the new generation of researchers and develop a culture that embraces open science.” Some suggestions about how these “appropriate support systems” would look would be very helpful. Similarly, where the authors state “A concerted approach is needed from all stakeholders including research funders, university and institutional management and researchers to actively improve how research is valued, how career progression is evaluated and to explicitly seek engagement in open science activities,” some specific suggestions would be extremely helpful.

 In addition, I have a number of minor comments about the wording below:

 Abstract:

 Background:

 I would change the wording to “Limited research has been conducted on the barriers and enablers to practicing open science for early career researchers.”

 Introduction:

 This is extremely well written and concisely sets out the background to the study.

 Qualitative data collection:

 Change wording from “over the phone” to “by telephone”

 Change wording from “and were facilitated” to “and interviews were facilitated”.

 Table 1, footnote.

 Change “multiple-choice answers” to “more than one answer”

*The ‘what’ of open science*

 I would remove the last sentence of the quote, from “So yeah ….”

*The “why” of open science*

 “think more thoroughly…” should be changed to “thinking more thoroughly”.

 P7, bottom: Change “…on the opposite hand” to “on the other hand”.

 P9, top:

 Should read “there is a responsibility for researchers …”

 Strengths and Weaknesses:

 Last sentence: Should read “The rigour with …”

Is the work clearly and accurately presented and does it cite the current literature?

Yes

If applicable, is the statistical analysis and its interpretation appropriate?

Yes

Are all the source data underlying the results available to ensure full reproducibility?

Partly

Is the study design appropriate and is the work technically sound?

Yes

Are the conclusions drawn adequately supported by the results?

Yes

Are sufficient details of methods and analysis provided to allow replication by others?

Yes

Reviewer Expertise:

Qualitative methodology; Mixed methods.

I confirm that I have read this submission and believe that I have an appropriate level of expertise to confirm that it is of an acceptable scientific standard, however I have significant reservations, as outlined above.

# Response

**Published:** 2021-01-14

**Authors:** Elaine Toomey

**Affiliations:** University of Limerick, Ireland

**REVIEWER 2**

 Comment 1: *My only criticism is that the suggestions for how to address the barriers mentioned in the article are not very specific. For example, the authors state: “It is clear that appropriate support systems are needed to help ECRs to become the new generation of researchers and develop a culture that embraces open science.” Some suggestions about how these “appropriate support systems” would look would be very helpful. Similarly, where the authors state “A concerted approach is needed from all stakeholders including research funders, university and institutional management and researchers to actively improve how research is valued, how career progression is evaluated and to explicitly seek engagement in open science activities,” some specific suggestions would be extremely helpful.*

 Response 1: Thank you for your comments. We have revised our discussion and conclusions to include a specific section on implications for practice, policy and research on p18-19 (specific additions highlighted below in intalics). This includes specific suggestions on what appropriate support systems could be developed, as well as specific suggestions regarding the concerted approach from all stakeholders.

***Implications for Practice, Policy and Research***

 It is clear that appropriate support systems are needed to help ECRs to become the new generation of researchers and develop a culture that embraces open science. *For example, our participants emphasised the importance of the availability of training events, education and resources within research active institutions to improve open science awareness, knowledge and skills for researchers. However, both formal and informal undergraduate and postgraduate research training and supervision in these institutions should also endeavour to include a holistic focus on the bigger picture beyond knowledge and skills, e.g., by focusing on the benefits and impact of transparent and better quality health research on patients and society, in essence to intrinsically motivate ECRs to practice open science behaviours. Moreover, both ‘top down’ and ‘bottom up*’ approaches are needed from all stakeholders including research funders, university and institutional management and individual researchers *at all levels (e.g. principal investigators, supervisors, ECRs) to facilitate the development of a research culture of transparency and openness. Specifically, as advocated for by Moher and colleagues, research active institutions and governing bodies should revisit the policies and metrics by which research and its outputs are measured, and accordingly how researchers are hired, promoted and evaluated* and to explicitly seek engagement in open science activities *(Moher et al., 2020)*. *To support this, institutions could consider the appointment of specific open science officers or champions, or establishing open research committees that consider beyond just open data or open access publishing, but the entire research cycle, as well as building an open research culture.*

*For ECRs, our study highlights some substantial challenges to practicing open science, but with several opportunities also identified for ‘bottom up’ solutions and strategies to overcome and address these. First, ECRs can ‘get active’ by seeking out others interested in practicing open science and building an open science community through grassroots initiatives like ReproducibiliTea journal clubs, or Open Science Cafés, and build themselves a platform to be heard. Second, ECRs can ‘get vocal’ by using these platforms to lobby and engage with institutional management like ethics committees around data sharing, libraries regarding open access and open publishing policies, and university promotion committees. Third, ECRs can ‘get inquisitive’ by conducting research on research that would facilitate and promote open research practices, such as addressing sensitivities of data sharing and identify evidence-based ways of balancing ethics with data transparency*. Further research is also needed to explore, develop, implement and evaluate the impact of different initiatives and interventions, such as the Framework for Open and Reproducible Research Training (FORRT) and Facilitate Open Science Training for European Research (FOSTER) (Pontika et al., 2015) and *Principles and Practices of Open Research: Teaching, Research, Impact, and Learning (PaPOR TRaIL)(Egan et al., 2020)*, at different levels (e.g. undergraduate, postgraduate, postdoctoral) to help overcome the obstacles to open science faced by ECRs working in health research and across other disciplines and *improve open science practices.*

 Comment 2: *In addition, I have a number of minor comments about the wording below:*

 Abstract: *I would change the wording to “* *Limited research has been conducted on the barriers and enablers to practicing open science for early career researchers* *.”*

 Response 2: This has been changed on p2 (abstract).

 Comment 3 *: Qualitative data collection: Change wording from “over the phone” to “by telephone”*

*Change wording from “and were facilitated” to “and interviews were facilitated”.*

 Response 3: These changes have been made on p5.

 Comment 4: *Table 1, footnote. Change “multiple-choice answers” to “more than one answer”*

 Response 4: This change has been made on p8.

 Comment 5: *The ‘what’ of open science: I would remove the last sentence of the quote, from “So yeah ….”*

 Response 5: Quote INT 1 page 10: the last sentence *‘* *So yeah it really just. I just thought it was kind of open access publications'* has been removed. 

 Comment 6: *The “why” of open science: “think more thoroughly…” should be changed to “thinking more thoroughly”.*

 Response 6: This change has been made on p10.

 Comment 7: *P7, bottom: Change “…on the opposite hand” to “on the other hand”.*

 Response 7: This change has been made on p13 (p7 as per online published version)

 Comment 8: *P9, top: Should read “there is a responsibility for researchers …”*

 Response 8: This change has been made on p15 (p9 as per online published version)

 Comment 9: *Strengths and Weaknesses: Last sentence: Should read “The rigour with …”*

 Response 9: This change has been made on p19 (p10 as per online published version).

# Peer review: Reviewer response for version 1

**DOI:** 10.21956/hrbopenres.14227.r27890

**Published:** 2020-09-22

**Authors:** Joshua Polanin

**Affiliations:** 1 Research & Evaluation, American Institutes for Research, Washington, DC, USA

Thank you for the opportunity to review this manuscript. As the authors know, I'm a big proponent of open science and the open science movement. So I am particularly pleased to see this issue being addressed with ECR. 

 In this article, the authors conducted a mixed methods research study, interviewing 14 students and also asking them to complete a survey. The results indicated that the students support OS practices but some barriers - particularly institutionally or socially - may prevent their use.

 There's much to like in this article and frankly little to critique. Given its qualitative nature, the sample and sampling design seem fine. The authors clearly have developed the themes and the analysis process seemed sound. I enjoyed reading some of the quotes, too. One small critique - some of the quotes could probably be "cleaned up" a bit. Not changed substantively - but sometimes our spoken words don't always convey exactly what the written word tries to. So it might be useful to clean them up some. 

 So the biggest critique I can surmise is simply with the conclusions and discussion. I'd like to see the authors try and provide even greater suggestions about how to address the barriers or issues the respondents raised. How will we go from the start of the OS movement - where we are now - to the next generation? How can we ask ECRs to do more and be better than the last generation - what will it take? I'm not asking for world-breaking ideas, but a few more suggestions, written plainly and in its own section at the end, might go a long way here. 

 I appreciate the authors hard work on this manuscript. I look forward to reading it again soon.

Is the work clearly and accurately presented and does it cite the current literature?

Yes

If applicable, is the statistical analysis and its interpretation appropriate?

Yes

Are all the source data underlying the results available to ensure full reproducibility?

Partly

Is the study design appropriate and is the work technically sound?

Yes

Are the conclusions drawn adequately supported by the results?

Yes

Are sufficient details of methods and analysis provided to allow replication by others?

Yes

Reviewer Expertise:

Quantitative methodology; meta-analysis; open science practices.

I confirm that I have read this submission and believe that I have an appropriate level of expertise to confirm that it is of an acceptable scientific standard, however I have significant reservations, as outlined above.

# Response

**Published:** 2021-01-14

**Authors:** Elaine Toomey

**Affiliations:** University of Limerick, Ireland

**REVIEWER 1:**

 Comment 1: *One small critique - some of the quotes could probably be "cleaned up" a bit. Not changed substantively - but sometimes our spoken words don't always convey exactly what the written word tries to. So it might be useful to clean them up some.*

 Response 1: Thank you for taking the time to review our manuscript and for your helpful comments. We have cleaned up the quotes as outlined below:

 Quote INT 1 page 10: the last sentence *‘* *So yeah it really just. I just thought it was kind of open access publications'* has been removed. 

 Quote INT 10 page 10: the sentence ‘ *So it’s about having better research really. It’s kind of openness'* has been removed.

 Quote INT 10 page 11: the sentence *'and spending time on the protocol'* has been removed

 Quote INT 14 page 11: the sentence *'And this is a perfect example of where everybody can benefit from this*' has been removed

 Quote INT 12 page 12: the sentence *'So again I see that as a similar challenge as well. So yeah no I don’t think so*' has been removed

 Quote INT 2 page 15: the sentence *'in a couple of instances, not in all the instances' *has been removed

 Quote INT 6 page 16: the sentence *'if you’re really interested like say it wasn’t in your research group'* has been removed

 Quote INT 4 page 16: the sentence ' *and any of that that you could put stuff out there' *has been removed

 Comment 2: *So the biggest critique I can surmise is simply with the conclusions and discussion. I'd like to see the authors try and provide even greater suggestions about how to address the barriers or issues the respondents raised. How will we go from the start of the OS movement - where we are now - to the next generation? How can we ask ECRs to do more and be better than the last generation - what will it take? I'm not asking for world-breaking ideas, but a few more suggestions, written plainly and in its own section at the end, might go a long way here.*

 Response 2: Thank you for this suggestion. We have revised our discussion and conclusions to include a specific section on implications for practice, policy and research on p18-19 (specific additions highlighted below in italics). This includes a specific section for ECRs in relation to approaches they can take to move open science practices forwards.

***Implications for Practice, Policy and Research***

 It is clear that appropriate support systems are needed to help ECRs to become the new generation of researchers and develop a culture that embraces open science. *For example, our participants emphasised the importance of the availability of training events, education and resources within research active institutions to improve open science awareness, knowledge and skills for researchers. However, both formal and informal undergraduate and postgraduate research training and supervision in these institutions should also endeavour to include a holistic focus on the bigger picture beyond knowledge and skills, e.g., by focusing on the benefits and impact of transparent and better quality health research on patients and society, in essence to intrinsically motivate ECRs to practice open science behaviours. Moreover, both ‘top down’ and ‘bottom up*’ approaches are needed from all stakeholders including research funders, university and institutional management and individual researchers *at all levels (e.g. principal investigators, supervisors, ECRs) to facilitate the development of a research culture of transparency and openness. Specifically, as advocated for by Moher and colleagues, research active institutions and governing bodies should revisit the policies and metrics by which research and its outputs are measured, and accordingly how researchers are hired, promoted and evaluated* and to explicitly seek engagement in open science activities *(Moher et al., 2020)*. *To support this, institutions could consider the appointment of specific open science officers or champions, or establishing open research committees that consider beyond just open data or open access publishing, but the entire research cycle, as well as building an open research culture.*

*For ECRs, our study highlights some substantial challenges to practicing open science, but with several opportunities also identified for ‘bottom up’ solutions and strategies to overcome and address these. First, ECRs can ‘get active’ by seeking out others interested in practicing open science and building an open science community through grassroots initiatives like ReproducibiliTea journal clubs, or Open Science Cafés, and build themselves a platform to be heard. Second, ECRs can ‘get vocal’ by using these platforms to lobby and engage with institutional management like ethics committees around data sharing, libraries regarding open access and open publishing policies, and university promotion committees. Third, ECRs can ‘get inquisitive’ by conducting research on research that would facilitate and promote open research practices, such as addressing sensitivities of data sharing and identify evidence-based ways of balancing ethics with data transparency*. Further research is also needed to explore, develop, implement and evaluate the impact of different initiatives and interventions, such as the Framework for Open and Reproducible Research Training (FORRT) and Facilitate Open Science Training for European Research (FOSTER) (Pontika et al., 2015) and *Principles and Practices of Open Research: Teaching, Research, Impact, and Learning (PaPOR TRaIL)(Egan et al., 2020)*, at different levels (e.g. undergraduate, postgraduate, postdoctoral) to help overcome the obstacles to open science faced by ECRs working in health research and across other disciplines and *improve open science practices.*
